# Native T1 lowering in iron overload and Anderson Fabry disease; a novel and early marker of disease

**DOI:** 10.1186/1532-429X-15-S1-O71

**Published:** 2013-01-30

**Authors:** Daniel Sado, Steven K White, Stefan K Piechnik, Sanjay M Banypersad, Thomas A Treibel, Marianna Fontana, Gaby Captur, Viviana Maestrini, Robin Lachmann, Derralyn Hughes, Elaine Murphy, John Porter, Atul Mehta, Perry Elliott, James Moon

**Affiliations:** 1 The Heart Hospital, London, UK; 2 University College London, London, UK; 3 University of Oxford, Oxford, UK; 4 The National Hospital for Neurology and Neurosurgery, London, UK; 5 Royal Free Hospital, London, UK

## Background

T1 mapping is a powerful technique for ECV quantification; native T1 has been shown to increase in a variety of conditions including oedema, fibrosis and amyloid. Iron and fat lower T1. Anderson Fabry disease (AFD) is a fat storage disease, cardiac iron occurs in transfusion dependent patients. We hypothesised that T1 lowering would diagnose early cardiac involvement, track disease severity and discriminate from other mimic pathologies.

## Methods

280 subjects were studied: iron overload (n=53), AFD (n=44, 55% with LVH, all genotyped), healthy volunteers (HV, n=67, 0% with LVH), hypertension (HYP, n=41, 24% with LVH), hypertrophic cardiomyopathy (HCM, n=34, 100% with LVH), severe aortic stenosis (AS, n=21, 81% with LVH) and definite AL cardiac amyloidosis (AMY, n=20, 100% with LVH). Along with routine clinical CMR, native, non-contrast T1 mapping was performed using the Sh-MOLLI technique at 1.5T without gadolinium administration. T2*(iron overload) and LGE and LV mass (AFD and LVH diseases) were also assessed.

## Results

Compared to health volunteers, septal T1 was lower in iron overload and AFD and higher in other diseases (iron overload vs AFD vs healthy volunteers vs other patients, 836±138 ms, 882±47 ms, 968±32 ms, 1018±74 ms, P<0.0001).

**Figure 1:**
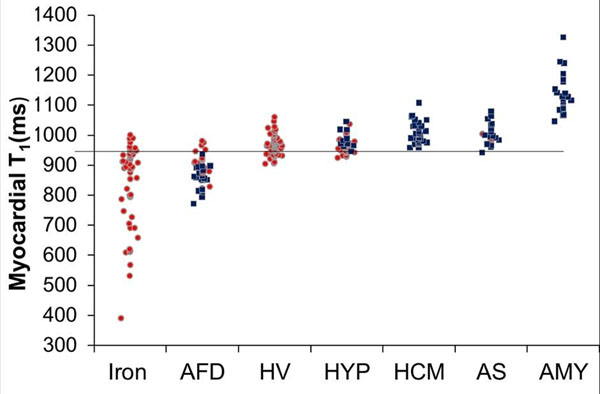
Septal T1 in all participants either with (filled blue square) or without (hollow red circle) LVH. The horizontal line at 940ms has no LVH AFD patients above it, and no LVH non-AFD patients below it.

In patients with LVH (n=105), T1 discriminated completely between AFD and all other diseases with no overlap (figure 1). In AFD, T1 correlated inversely with wall thickness (R=-0.51, P=0.0004) and was abnormal in 40% of subjects even without LVH. Segmentally, AFD showed pseudo-normalisation or elevation of T1 in the LV infero-lateral wall, the extent correlating with the presence or absence of post contrast late gadolinium enhancement (1001±82 ms vs 891±38 ms, P<0.0001).

**Figure 2:**
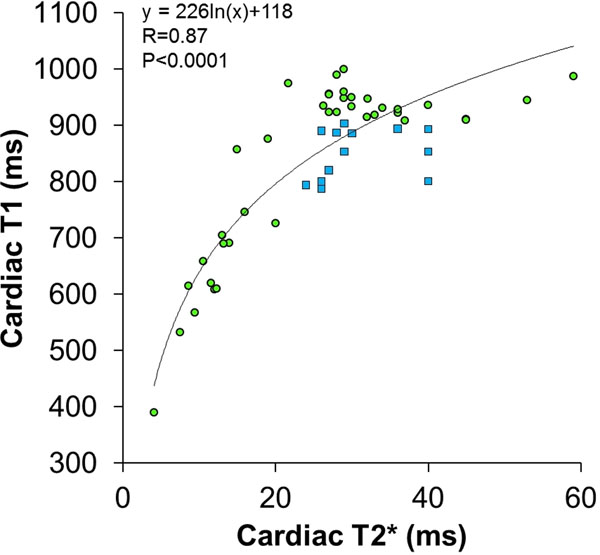
Cardiac septal T1 vs T2*. The filled blue square points show patients where the T1 was low despite a normal T2*. The filled green circular points show patients where T1 and T2* were both normal, or both abnormal.

In iron overload, myocardial T1 strongly correlated with T2* (R=0.87, P<0.0001, figure 2). No patient with low T2* had normal T1, but 25% cases characterised by a normal T2* (n=37) had low myocardial T1 (from 2 to 5 standard deviations below normal) suggesting a quarter of patients referred have mild iron loading despite a normal T2*.

## Conclusions

Impressive lowering of myocardial T1 can occur in AFD and particularly iron overloading. Unsuspected cardiac involvement was found in 40% of AFD patients without LVH and 25% of possible iron overload patients where T2*was normal. When compared to the common causes of LVH, the detection of T1 lowering appears definitively diagnostic of AFD.

## Funding

1) British Heart Foundation.

2) Genzyme Pharmaceuticals.

